# Potential confounding mutations in Keio knockout strains: implications for research accuracy

**DOI:** 10.1128/spectrum.02036-24

**Published:** 2025-03-31

**Authors:** Oishi Sen, Xianghui Liu, Staffan Kjelleberg, Scott A. Rice, Thomas Seviour

**Affiliations:** 1 Singapore Centre for Environmental Life Sciences Engineering, Nanyang Technological University 548257 , Singapore, Singapore; 2 School of Biological Sciences, Nanyang Technological University 219572 , Singapore, Singapore; 3 The Australian Institute for Microbiology and Infection, University of Technology Sydney, Ultimo, Australia; 4 CSIRO, Agriculture and Food, Westmead and Microbiomes for One Systems Health 261802 , Canberra, Australia; 5 WATEC Aarhus University Centre for Water Technology, Universitetsbyen 559383 , Aarhus, Denmark; Indian Institute of Technology Hyderabad, Hyderabad, Telangana, India

**Keywords:** *Escherichia coli*, gene knockout, whole genome sequencing, genomic mutations

## Abstract

The Keio library of single-gene knock-out mutants of *Escherichia coli* is useful for the research community. It has been used to analyze the role of various *E. coli* genes in alcohol tolerance, multi-drug resistance, and biofilm formation. The current study provides a general overview of potential single nucleotide polymorphisms (SNPs), insertion–deletion of bases (≤50 nucleotides, INDELs) in the genome of a set of 21 knock-out mutants of the Keio collection in comparison to the parent strain. A small number of SNPs and INDELs were predicted in the coding and intergenic regions of the knock-out mutants in comparison to the parental strain through sequencing and bioinformatic analysis. Mutations in the coding regions of genes (different from the actual gene knocked out in the mutants) led to different types of mutations in the affected genes, ranging from nonsense mutations to frameshift mutations, which could affect the functionality of the resulting gene products. These mutations in the intergenic and coding regions could lead to phenotypic differences in the single-gene knock-out mutant strains in comparison to the parent strain, independent of the desired gene deletion. This, in turn, could be misinterpreted by researchers using these strains as differences caused by the missing gene. While this is a preliminary study based on only 21 strains of the Keio collection, the deleted genes in the mutants used in this study were approximately evenly distributed across the entire genome. This study likely indicates the possibility of such mutations in other Keio strains, although a larger sample size of knock-out mutants would be required to understand the likelihood of such mutations across the library.

**IMPORTANCE:**

The Keio library of single-gene knock-out mutants of *Escherichia coli* has been widely used for a variety of studies. However, mutations might appear in the genome of these strains over time, leading to differences in the characteristics of the mutant and parent strains that are independent of the gene deletions of interest. This study predicts the presence of a few SNPs and INDELs in some of the knock-out mutants from the Keio collection, which could potentially alter the phenotypic attributes of the knock-out mutants with no role of the deleted gene towards this change. Therefore, this study highlights the possibility of the presence of such mutations in other strains of the library and the importance of conducting additional steps, such as complementation assays, to confirm the outcomes of studies comparing specific attributes of the knock-out mutants with the parental strain.

## INTRODUCTION

Knocking out non-essential genes from the parent bacterial strain offers a valuable way of studying the role of individual genes in bacterial fitness under various environmental conditions. However, the process of knocking out genes can be time-consuming. Thus, a comprehensive library of almost all non-essential, single-gene knock-out mutants from *Escherichia coli* BW25113 was developed and made available to the scientific community (i.e., the Keio collection) (1). This has subsequently been widely used for many biological studies (2–16), especially given that many fundamental biological processes are often first studied in *E. coli* as a model bacterium before extending understandings to other non-model bacteria or eukaryotes. For example, 84 genes were screened using knock-out mutants from the Keio collection to study their role in the butanol tolerance of *E. coli* (9). In another study, the Keio library was used to identify 1,564 chemical-genetic interactions for 15 antibiotics in *E. coli*, which revealed common genes possibly involved in multidrug resistance (10). The knock-out strains from the Keio collection have also been used to investigate the involvement of specific genes in biofilm formation (11, 12), ethanol tolerance (13), zinc resistance (14), colistin sensitivity (15) and boric acid resistance (16).

However, it has been more than 17 years since this library was first made. Sub-culturing of these strains may result in the introduction of spontaneous mutations in the genome of the single-gene knock-out mutants, which might differ from the parental strain. Such mutations could, in turn, confound attributing phenotypes to the gene deletions of interest. Therefore, in this study, we subjected 21 single-gene knock-out mutants from the Keio collection to whole genome sequencing and compared the results with the parental strain, which was also re-sequenced here, to describe the presence and distribution of single nucleotide polymorphisms (SNPs) and insertions and deletions that are ≤50 nucleotides (INDELs) using breseq software (17).

## RESULTS AND DISCUSSION

### The genomes of the single-gene knock-out mutants confirmed the absence of the respective genes of interest

For the development of the single-gene knock-out mutants, the coding regions of the genes of interest were replaced by a kanamycin cassette, leading to their inactivation (1). The locations of the deleted genes in the knock-out mutants used in this study were approximately evenly distributed across the entire genome (Fig. S1), with the exception of a few genes occupying the same operons (such as *ΔrcsB/C/D, ΔacrA/B, ΔenvZ/OmpR*, and *ΔcpxA/P/R*). These were assessed to determine whether gene deletions belonging to the same operon or similar genomic locations lead to similar genomic mutations. Knock-out mutants *ΔompC* and *ΔrcsD* with deleted genes belonging to different operons but very close in their genomic locations were also analysed. The deleted genes in the knock-out mutants used in this study encoded different types of proteins, such as cytoplasmic, periplasmic, inner membrane, and outer membrane proteins (Table S1), and the genomic mutations were compared between knock-out mutants lacking proteins from the same cellular locations (such as the periplasm, cytoplasm, inner membrane, or outer membrane). All 21 knock-out mutant strains of the Keio collection used in the present study (Table S1) were verified by whole genome sequencing, which confirmed that the genes of interest were indeed deleted. When the rest of the genome was compared with the parental sequence (NCBI accession: CP009273.1), the mutations predicted by breseq in the single-gene knock-out mutant strains are described in Table 1.

**TABLE 1:** Mutation predictions by breseq (one-letter abbreviations are used for the amino acids)

Strain	Mutation	Genomic position	Annotation^*a*^	Gene
Δ*acrA*	C→G	434,596	M346I (ATG→ATC), missense mutation	*dxs*
	Insertion of 1,199 bp	2,400,257	Intergenic (-137/–780), IS5 mediated	*lrhA*/*alaA*
Δ*acrB*	C→T	3,941,103	W75* (TGG→TGA), nonsense mutation	*hdfR*
Δ*cpxA*	A→T	435,152	F161Y (TTT→TAT), missense mutation	*dxs*
	G→T	1,293,170	F136L (TTC→TTA), missense mutation	*adhE*
	T→C	2,719,426	Intergenic (−321/+2)	*kgtP*/*rrfG*
Δ*cpxP*	Insertion of 1,199 bp	1,972,818	Intergenic (-364/–413), IS5 mediated	*flhD*/*uspC*
Δ*cpxR*	Deletion of 1 bp	446,710	Coding (357/948 nt), frameshift mutation	*cyoA*
	Insertion of 1,199 bp	1,972,967	Intergenic (-513/–264), IS5 mediated	*flhD/uspC*
	T→C	2,719,426	Intergenic (−321/+2)	*kgtP*/*rrfG*
Δ*envZ*	T→C	2,719,426	Intergenic (−321/+2)	*kgtP*/*rrfG*
	Deletion of 6 bp	3,941,538	Coding (93–98/339 nt), non-frameshift mutation	*yifE*
Δ*ompA*	Insertion of 1,199 bp	1,972,967	Intergenic (-513/–264), IS5 mediated	*flhD*/*uspC*
Δ*ompF*	Insertion of 6 bp	3,046,837	Coding (41/1179 nt), non-frameshift mutation	*ubiH*
Δ*ompR*	G→A	2,400,090	L11F (CTC→TTC), missense mutation	*lrhA*
	T→C	2,719,426	Intergenic (−321/+2)	*kgtP*/*rrfG*
Δ*ompT*	G→A	4,036,889	A38T (GCG→ACG), missense mutation	*dsbA*
Δ*ompX*	T→C	2,719,426	Intergenic (−321/+2)	*kgtP*/*rrfG*
	G→T	4,259,218	Intergenic (−278/–124)	*yjbS*/*aphA*
Δ*rcsB*	C→T	170,655	V239M (GTG→ATG), missense mutation	*hemL*
	T→C	2,719,426	Intergenic (−321/+2)	*kgtP*/*rrfG*
Δ*rcsC*	T→C	2,719,426	Intergenic (−321/+2)	*kgtP*/*rrfG*
	Insertion of 1,199 bp	3,797,089	Coding (406–409/1080 nt), frameshift mutation, IS5 mediated	*waaB*
Δ*rcsD*	T→C	2,719,426	Intergenic (−321/+2)	*kgtP*/*rrfG*
Δ*soxS*	T→C	2,719,426	Intergenic (−321/+2)	*kgtP*/*rrfG*
	T→G	3,940,652	T226P (ACC→CCC), missense mutation	*hdfR*
Δ*spy*	T→C	2,719,426	Intergenic (−321/+2)	*kgtP*/*rrfG*
	G→A	3,085,323	Q29Q (CAG→CAA), silent mutation	*gshB*

^*a*^ / Intergenic region in between two genes, → Substitution of one base with another, − Number of bp upstream of the gene, + Number of bp downstream of the gene.

### A small number of SNPs/INDELs were predicted in some knock-out mutants

**Fig 1:**
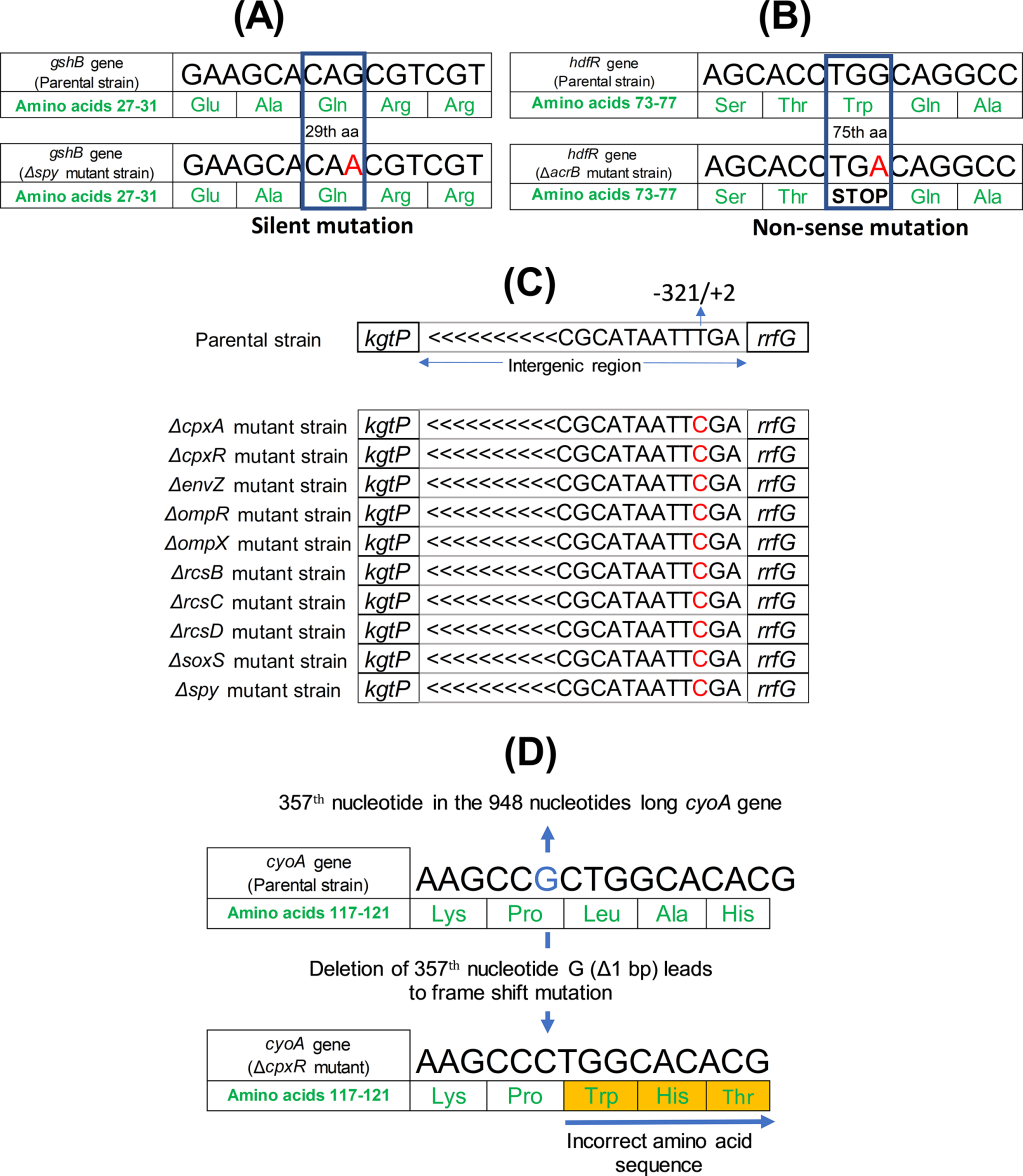
Red color denotes the modified base in A, B, and C. (**A**) A silent mutation in the *gshB* gene of the *Δspy* mutant will likely have no functional impact on the encoded protein, and (**B**) a nonsense mutation in the *hdfR* gene of the *ΔacrB* mutant could result in a truncated protein, affecting its functionality. (**C**) The same SNP was found at the same position in the intergenic region between *kgtP* and *rrfG* genes in *ΔcpxA, ΔcpxR, ΔenvZ, ΔompR, ΔompX, ΔrcsB, ΔrcsC, ΔrcsD, ΔsoxS,* and *Δspy* mutants. (**D**) A frameshift mutation observed in the *cyoA* gene of the *ΔcpxR* mutant would likely lead to gene inactivation or non-functional protein.

There were no SNPs or INDELs detected in the resequenced parental strain, Δ*degP, ΔlamB, ΔompC, ΔfadL,* and Δ*tolC* mutants in comparison to the reference parental genome in the mutation predictions by breseq (Table 1). Overall, the number of SNPs or INDELs found in the mutant strains compared with the parental strain was quite low. The breseq results indicated the presence of one SNP in one of the coding regions of Δ*acrA,* Δ*acrB,* Δ*ompR,* Δ*ompT,* Δ*rcsB,* Δ*soxS,* and Δ*spy* mutants*,* and the presence of two SNPs in the coding regions of the Δ*cpxA* mutant (Table 1). Out of these SNPs, the SNP in the *gshB* gene of the Δ*spy* mutant is an example of a silent mutation (Fig. 1A) since the codon resulting from the mutation (CAA) codes for the same amino acid as the codon before the mutation (CAG), i.e., glutamine (Q). A nonsense mutation was observed in the *hdfR* gene of the Δ*acrB* mutant (Fig. 1B), which codes for the *flhDC* operon transcriptional repressor HdfR (18). In the current study, the SNP found in the gene *hdfR* of the Δ*acrB* mutant would result in the replacement of the codon (TGG) for tryptophan (W) in the 75th amino acid position of the protein with a stop codon (TGA). This would generate a truncated protein with only 74 amino acids (aa) instead of 279 aa for the native gene. It is reported that a loss of function mutation in the *hdfr* gene increases the availability of NADPH in the parent strain *E. coli* BW25113, probably because HdfR facilitates the expression of enzymes involved in NADPH-dependent glutamate synthesis (19).

Interestingly, an SNP (T→C) in the same genomic position was found in the Δ*cpxA*, Δ*cpxR*, Δ*envZ*, Δ*ompR*, Δ*ompX*, Δ*rcsB*, Δ*rcsC*, Δ*rcsD*, Δ*soxS,* and Δ*spy* mutants (Fig. 1C). However, this nucleotide falls in the intergenic region in between genes *kgtP* and *rrfG*. Therefore, the SNP does not alter the proteins encoded by the flanking genes. Similarly, another SNP was found in the intergenic region in between genes *yjbS* and *aphA* in the Δ*ompX* mutant (Table 1). While these are unlikely to alter the encoded proteins, mutations in the intergenic regions might affect the expression of nearby genes or non-coding regulatory RNA molecules (20). It is reported that intergenic mutations in bacteria may alter the transcription level of genes and may contribute to the evolution of bacterial traits, such as antibiotic sensitivity (21) and adaptation to the gut environment (22).

**Fig 2:**
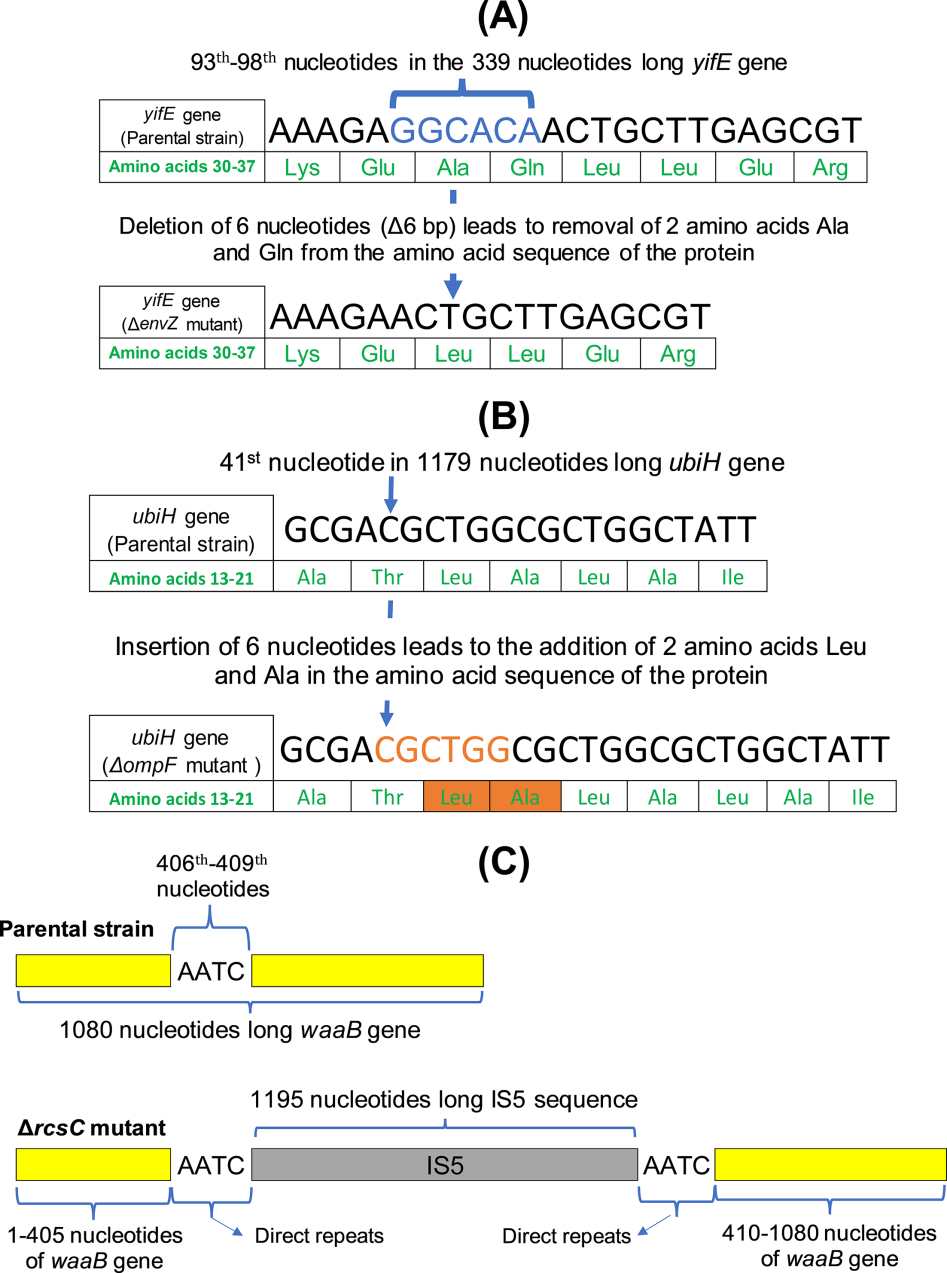
(**A**) Deletion of six nucleotides (93rd–98th nucleotides) from the *yifE gene* of *ΔenvZ* mutant would result in the loss of two amino acids (32nd and 33rd amino acids) from the protein. The deleted bases in the *ΔenvZ* mutant are shown in blue in the parental strain. (**B**) Insertion of six nucleotides in the coding region of the *ubiH* gene in the *ΔompF* mutant would result in the addition of two amino acids to the protein. The inserted sequence and the two newly incorporated amino acids in the *ΔompF* mutant are colored in orange. (**C**) Insertion of IS5 (1,195 bp) + direct repeat (4 bp) after the 409th nucleotide in the *waaB* gene (colored in yellow) of *ΔrcsC* mutant would likely lead to frameshift mutation and gene inactivation.

Sequence analysis indicated the deletion of one nucleotide from the coding region of the *cyoA* gene in the Δ*cpxR* mutant. This would lead to a frameshift mutation in the gene (Fig. 1D). In the Δ*envZ* mutant, a deletion of six bp was observed in the *yifE* coding region. This deletion would result in the loss of two amino acids from the protein (Fig. 2A). Similarly, the insertion of 6 nucleotides in the coding region of the *ubiH* gene in *ΔompF* mutant would lead to the addition of two amino acids to the protein (Fig. 2B). One transposon-mediated insertion mutation was observed in *ΔacrA, ΔcpxA, ΔcpxP, ΔcpxR, ΔompA,* and *ΔrcsC* mutants (Table 1). All of these insertions were mediated by IS5. The transposon-mediated insertions were in the intergenic region of the knock-out mutants, except the one found in the *ΔrcsC* mutant. The transposon-mediated insertion in the *ΔrcsC* mutant occurs in the coding region of the *waaB* gene, which codes for lipopolysaccharide 1,6-galactosyltransferase (18). The insertion, which is 1,195 (IS5) + 4 nucleotides (direct repeat) long, occurs after the 409th nucleotide of the 1,080 nt long *waaB* gene and, most likely, would lead to frameshift mutation and inactivation of the gene (Fig. 2C).

No similarity was observed in the pattern of SNPs in the mutant strains with deleted genes belonging to the same operon, such as *ΔrcsB/C/D, ΔacrA/B, ΔEnvZ/OmpR,* and *ΔcpxA/P/R* (Table 1). Likewise, no similarity in mutation patterns was observed between *ΔompC* and *ΔrcsD* mutants (i.e., different operons but similar genomic location). The same SNP in the intergenic region between *kgtP* and *rrfG* genes (Fig. 1C) was found in different mutant strains, with gene deletions from different operons and genomic locations. The IS5-mediated insertion was predicted in the same intergenic region between genes *flhD* and *uspC* (Table 1) in *ΔcpxP* and *ΔcpxR* mutants *(*with deleted genes from the same operon). However, an IS5-mediated insertion in the same intergenic region was also predicted in the *ΔompA* mutant. Therefore, the mutations predicted in this study were likely not influenced by the genomic locations/operon of the genes deleted. No notable similarity in mutation patterns was observed between knock-out mutants lacking proteins belonging to the same cellular locations.

The development of mutations in *E. coli* over time in a laboratory environment is not uncommon. In a long-term evolution experiment where *E. coli* was maintained in a simple lab environment, *E. coli* continued to improve their fitness despite being in the same environment for >35 years (23). Whole genome sequencing suggested the appearance of several SNPs and INDELs in the *E. coli* strains after 20 years of cultivation in a laboratory environment (24). However, all the mutations predicted in this study should be confirmed through phenotypic studies to understand if they have any impact on gene function or expression. Future studies might consider testing the phenotypic effect or functional impacts of such mutations via genetic manipulation and phenotypic assays. Nonetheless, the results in the current study highlight the importance of carrying out additional experiments, such as complementation experiments, to validate phenotypic observations gleaned from Keio strains. While this should be considered standard, conclusions are sometimes drawn from Keio mutant strains without such additional validation studies. Therefore, our observations will serve as a timely reminder to researchers to consider SNPs and INDELs when assigning phenotypic changes to knocked out genes. This study will likely raise awareness in the scientific community about this issue.

The single gene knock-out mutant library of *E. coli* is an invaluable resource, enabling rapid screening of the effect of individual gene deletions across a range of applications. It is cheap, convenient, and time-saving for the research community. Here, we provide preliminary studies indicating a small number of SNPs and INDELs in a subset of knock-out strains of the Keio collection in comparison to the parent strain. These mutations in the intergenic and coding regions could potentially lead to phenotypic differences in the mutant strains compared with the parent strain that are independent of the desired gene deletion and therefore could mislead studies comparing specific phenotypes between the knock-out strains and the parent strain. This problem has been overcome in various studies by expressing the deleted gene in the knock-out mutants (complementation experiments) and cross-checking if the desired or missing phenotype is recovered when the gene is expressed (14–16, 25, 26). However, this might not always work since single-gene deletions also affect the expression levels of downstream genes belonging to the same operon known as the polar effects (27). In such cases, complementation cannot restore the indirect effects of gene deletion. For instance, it is reported that the UV sensitivity of the *ΔybaB* mutant is not due to the absence of YbaB but due to lower expression of adjacent gene *recR* as an indirect effect of *ybaB* gene deletion (27). Therefore, in such cases, the genes downstream to the deleted gene and belonging to the same operon also need to be analyzed through gene knock-out and complementation studies.

While the current study involving 21 knock-out mutant strains from a library of 3,985 mutants (1) is not sufficient to draw a definitive conclusion about the entire Keio library, the location of the knocked out genes in the mutants used in this study are approximately evenly distributed across the entire genome representing the absence of genes from different genomic locations across the library. Moreover, these knocked out genes encode proteins belonging to the cytoplasm, periplasm, and inner or outer membrane, representing the absence of proteins from different locations of the cell. Mutation predictions by breseq indicated some form of mutation (SNPs/INDELs/IS5-mediated mutations) in 16 out of 21 mutants tested. Therefore, our findings on this subset of the Keio collection suggest the possibility of such mutations in other knock-out strains of the library. While this study suggests potential risks, this study should be expanded to a larger subset of the Keio collection to obtain a more robust numerical or statistical assessment of the likelihood of such mutations across the library.

## MATERIALS AND METHODS

### Strains and culture medium used in this study

All the strains listed in Table S1 were obtained from NBRP (NIG, Japan): *E. coli*. All the strains were grown in BD Difco Luria–Bertani Broth (Lennox) with the following composition: 10 g/L tryptone, 5 g/L yeast extract, and 5 g/L NaCl. The knock-out mutants were grown in the presence of 30 mg/mL kanamycin.

### Genomic DNA isolation

The genomic DNA of the knock-out mutants and their parent strain *E. coli* BW25113 (listed in Table S1) was isolated using the Invitrogen PureLink genomic DNA extraction kit according to the manufacturer’s instructions. The quality of the isolated genomic DNA was analyzed on 0.8% agarose gel to ensure the genomic DNA was intact and without any RNA contamination. The concentration of the genomic DNA was measured by Qubit dsDNA High Sensitivity (HS) Assay Kit.

### Whole genome sequencing

The genomic DNA samples were sequenced on Illumina MiSeq V3 Run with 301 bp paired-end sequences, which was carried out by the SCELSE sequencing facility. One sequencing library was produced from each sample, and the sequencing was performed in paired-end mode, yielding two reads (R1: read 1 and R2: read 2) for each sample. The sequences (fastq files) can be found at the NCBI sequence-read archive (https://www.ncbi.nlm.nih.gov/sra/PRJNA1095198).

### QC and reads trimming

The quality of the Illumina pair-ended reads was first assessed using FastQC version (28). Illumina reads were processed to cut the adaptor and quality-trimmed using Trimmomatic version 0.38 (29).

### Variant calling

Software breseq version 0.35.5 (17) was used, with default parameters, to map merged trimmed reads and identify mutations. *E. coli* BW25113 genome (NCBI accession: CP009273.1) was used as the reference for all the samples, and the mutations were then determined by comparing the knock-out strains with the parent strain.

## Data Availability

The sequences (fastq files) can be found at the NCBI sequence-read archive (https://www.ncbi.nlm.nih.gov/sra/PRJNA1095198).

## SUPPLEMENTAL MATERIAL

Supplemental material spectrum.02036-24-s0001.docx Fig. S1; Table S1.

ASM does not own the copyrights to Supplemental Material that may be linked to, or accessed through, an article. The authors have granted ASM a non-exclusive, world-wide license to publish the Supplemental Material files. Please contact the corresponding author directly for reuse.

## Abbreviations

SNPs: single nucleotide polymorphisms
INDELs: insertions and deletions that are ≤50 nucleotides
bp: base pair
nt: nucleotide
aa: amino acids
dsDNA: double stranded DNA
HS: High Sensitivity
