# Supplementary material for: Potential confounding mutations in Keio knockout strains: implications for research accuracy
Source: Microbiol Spectr. 2025 Mar 31;13(5):e02036-24. doi: 10.1128/spectrum.02036-24 (PMC12054083; doi:10.1128/spectrum.02036-24)
Supplement: Supplemental material — Fig. S1; Table S1. [file spectrum.02036-24-s0001.docx]

**Figure S1:** The *E. coli* Bw25113 genome is 4631469 bp long. Location of the deleted genes in the knock-out mutants used in this study are approximately evenly distributed across the genome. Here, 1-21 in the x-axis represents the following mutants: 1-*ΔdegP,* 2-*ΔacrB,* 3-*ΔacrA,* 4-*ΔompT,* 5-*ΔompX,* 6-*ΔompF,* 7-*ΔompA*, 8-*Δspy,* 9-*ΔompC,* 10-*ΔrcsD,* 11-*ΔrcsB,* 12-*ΔrcsC,* 13-*ΔfadL,* 14-*ΔtolC,* 15-*ΔenvZ,* 16-*ΔompR,* 17-*ΔcpxA,* 18*-ΔcpxR,* 19-*ΔcpxP,* 20-*ΔlamB,* 21-*ΔsoxS*

**Table S1: Strains used in this study**

| **Strain** | **Strain ID** | **Cellular location of encoded proteins of deleted genes** |
| --- | --- | --- |
| Resequenced parental strain | *E. coli* BW25113 | Not applicable |
| Δ*acrA* | JW0452-KC | Inner membrane |
| Δ*acrB* | JW0451-KC | Inner membrane |
| Δ*cpxA* | JW3882-KC | Inner membrane |
| Δ*cpxP* | JW5558-KC | Periplasm |
| Δ*cpxR* | JW3883-KC | Cytoplasm |
| Δ*degP* | JW0157-KC | Periplasm |
| Δ*envZ* | JW3367-KC | Inner Membrane |
| Δ*fadL* | JW2341-KC | Outer Membrane |
| Δ*lamB* | JW3996-KC | Outer Membrane |
| Δ*ompA* | JW0940-KC | Outer Membrane |
| Δ*ompC* | JW2203-KC | Outer Membrane |
| Δ*ompF* | JW0912-KC | Outer Membrane |
| Δ*ompR* | JW3368-KC | Cytoplasm |
| Δ*ompT* | JW0554-KC | Outer Membrane |
| Δ*ompX* | JW0799-KC | Outer Membrane |
| Δ*rcsB* | JW2205-KC | Cytoplasm |
| Δ*rcsC* | JW5917-KC | Inner Membrane |
| Δ*rcsD* | JW2204-KC | Inner Membrane |
| Δ*soxS* | JW4023-KC | Cytoplasm |
| Δ*spy* | JW1732-KC | Periplasm |
| Δ*tolC* | JW5503-KC | Outer Membrane |
